# Fibrinous Concretions in the Ventricles of the Heart

**Published:** 1847-12

**Authors:** G. Sprague

**Affiliations:** Kalamazoo, Mich.


ARTICLE II.

*Fibrinous Concretions in the Ventricles of the Heart, extending into the Pulmonary Artery and Aorta, with extensive hepatization of both lungs.* By G. SPRAGUE, M. D., of Kalamazoo, Mich.

Some ten days since (Oct. 23,) I was summoned to visit Hydenburgh, about a mile from this place, by occupation a farmer, æt. 36. The patient was a stranger to me, having settled in this vicinity withing the past year, from the State of New York.

I learned by the wife of the patient, that he had been for some fifteen years, much troubled with nervous symptoms; but was repeatedly assured that no cough or other indication of chronic lung disease, had ever manifested itself.

It seemed that on the afternoon of Oct. 20, Mr. H. was in usual health, and during that afternoon underwent rather severe bodily exertion. Near daylight of the following morning he had occasion to get out of bed when in a state of cutaneous moisture. He was immediately taken chilly, which state continued during the forenoon of the 21st. The afternoon of the same day was ushered in by some fever, pain in the chest, very laborious and rapid respiration. On the morning of the 22d, there seemed some abatement of pain, &c., but in the afternoon of the same day, there was a return of all the distressing symptoms in an increased degree. It was at two o'clock of the following day, (23d,) that I was requested to visit the case. I found the patient to be a tall spare man, in whom the nervous and bilious temperaments were predominant. Countenance anxious; restless and impatient. Skin hot and dry; pulse full, incompressible and twisting. Percussion dull throughout the chest; respiratory murmur hardly perceptible; breathing very rapid and laborious; complained of pain in the head and left lung, near the spine, rather laucinating upon bodily motion; expectorated a small amount of sputa, a little tinged with blood. From a free orifice, I abstracted xxiv℥ of blood, which presented but little of the buffy coat. As the blood flowed, the skin became cool and moist, the pain ceased, the pulse came down to 90, soft and compressible. Breathing less laborious, and not so frequent. I followed the venesection by an Armstrong opiate, with as much ipecac as the stomach would retain, which was repeated in two hours. This had the desired effect, viz: to prevent reaction. The skin remaining moist, expectoration easy, though scant, and the pain not returning during the day.

He was put upon the sub. mur., combined with the compound extract of colocynth, to be given *pro re nata*—as also a mixture of Dover's powders, ipecac, and tartar. ant.; enough given each hour to induce nausea. These means were kept up during the afternoon and night of the 23d. On the following morning found my patient rather quiet, pulse soft and compressible; skin moist; expectorated a small amount of tenaceous sputa, a little tinged with blood; some pain in the chest, though slight. Directed a mustard plaster upon the back, and applied a blister upon the front of the chest, to the right of the heart—same prescriptions continued as before sufficient to keep the bowels loose, skin moist, &c. Alvine evacuations, bilious. Visited again on the evening of the same day, found my patient rather restless; skin and conjunctiva becoming very much charged with bile. Pulse still soft and compressible; breathing more hurried and difficult. Similar prescriptions were continued, to the foregoing, sufficient to keep up their several effects through the 25th. On visiting my patient on the

26th, found no yielding of the deposite of bile in the skin and conjunctiva; more restless, delirious; pulse occasionally intermitting and unsteady in its beat; cough a little tightened; skin still moist. He remained in this condition through the 26th, becoming more delirious, asserting that he should expire when morning came. About ten o'clock at night, I was requested to visit the patient. Found a gradual wasting of the vital forces. At this time, Dr. White, (recently of the army,) was called in council, who judged, as I had been induced to do, from the voice of the patient, that there was probably some chronic disease of the lungs of long standing. We left him with a mild prescription, for the night. At daylight of the following morning, after having become somewhat composed, he expired.

*Autopsie twenty-eight hours after death.* Present, Drs. White, Allen, Howard, and myself.—Body not much wasted, skin and eyes very yellow. Upon opening the thorax, there was found firm adhesions throughout the chest, between the two pleuræ. Pericardium contained some five or six ounces of fluid. Heart considerably hypertrophied, though firm in texture, and presenting in its external appearance considerable enlargement of its blood vessels. Upon opening the right ventricle, it was found to contain two fibrinous concretions. The base of one of them was about three-fourths of an inch in width, and one-eighth in thickness, gradually narrowing down to a point, and extending itself into the pulmonary artery. The length of this formation was about six inches. It had a fimbriated extremity, made up of firm blood vessels distinct from each other. Its base was attached very firmly by distinct blood vessels and fibres, to the columnæ comae. The second formation found in the same ventricle, arose by two bases, in the same manner as the first, each base being three-fourths of an inch in width, and one eighth in thickness, coming together at the entrance of the pulmonary artery, giving off a branch three eighths of an inch in width, reaching the bifurcation, and giving a branch to the right and left, of an inch in length, with fimbriated ends, as the other. Upon opening the left ventricle, a deposit of the same kind was found, with as distinct an organization, having a base one inch in width and one fourth in thickness, with several extremities having fimbriated ends, and reaching about four inches from its origin into the aorta. There was also a second formation commencing to organize in the same cavity of considerable size, though imperfect. Their texture is as firm as muscle, and the fibres as distinctly marked, though in color they are quite pale. Their entire weight was nine drachms. The entire body of the lungs were found engorged and hepatized, presenting much the appearance of diseased liver. In several places small abscesses were found, containing thin pus. The liver and spleen were a little engorged and enlarged, though presenting no very striking marks of disease. The gall bladder was distended with vitriated bile.

Dunglison records a case somewhat similar to the foregoing, though more limited in extent, and producing the same effects upon the lungs as were here produced, though the hepatization was confined to one lung. Under fibrinous concretions in the heart, that learned author remarks: In a case of great impediment to the circulation of the blood through the lungs, which fell under the author's care, and in which, on application of the stethescope, the respiratory murmur was totally inaudible in any part of the right lung, whilst it was distinct in the left; and percussion on the right side gave the dull, heavy sound of a solid mass—the sound of the left side of the heart was so loud as to countenance the idea of dilatation of the cavity with thinness of the parietes—whilst the sound on the right side was indistinct, but there was a peculiar *bruit*, appearing, as it were, compounded of the *bruit de soufflet*, or bellows sound, with the *freemissement cataive*, and evidently indicating the existence of some impediment to the passage of the blood through the right heart. On the dissection of this case, the right lung was found completely hepatized throughout its whole extent, whilst the left lung was nearly healthy. On taking out the heart, the right pulmonary artery was found completely plugged up by a same organized mass, having the appearance of muscle, extending through the semilunar valves, and attaching itself strongly to the columnæ comae of the right ventricle. The same author quotes the following from Hughes: Occasionally, the polyphoid and polypiform concretions produce death by blocking up one of the apertures of the heart. Watson, in his Practice of Physic, speaks of mechanical impediments to the passage of blood from the heart, being in many cases of a permanent character, and causing hypertrophy of the ventricles, but does not specify as to what these impediments are. We look in vain in Latham's clinic of the diseases of the heart, for any account of such formations. Professor Dunglison again says, (Cyclop. of Practical Medicine,) at a late clinical lecture at the Philadelphia Hospital, the writer exhibited to the class a semi-organized fibrinous concretion in the right ventricle, the existence of which he foretold before dissolution, owing to the sound of impeded transmission of blood through the heart, in a person dying of pleura pneumonia—and a similar case occurring in his service in the Baltimore Infirmary, was published by Dr. McNeal, in the American Medical Intelligencer, July 1st, 1837. Armstrong says: It was an opinion that polypi formed about the heart. In many bodies you will find strings of coagulable lymph hanging about the ventricles or auricles, or in the pulmonary artery or veins; they are formed during the agonies of death.

That the formations in the case here reported, did not take place in this manner, is very certain. Their firm, well developed, fibrinous organization, their distinct origin by blood vessels, and the distinctness and tenacity of the vascular, fimbriated extremities, as well as the condition of the lungs, all go to prove that a long period only had been sufficient to produce such a condition.

